# A case of COVID-19 complicated by massive gastrointestinal bleeding

**DOI:** 10.1093/gastro/goaa067

**Published:** 2020-12-10

**Authors:** Yan-Ling Wang, Jin-Song Mu, Xiao-Bao Qi, Wen-Hui Zhang

**Affiliations:** 1 Liver Cirrhosis Diagnosis and Treatment Center, 5th Medical Center of PLA General Hospital of China, Beijing, P. R. China; 2 Intensive Care Unit, 5th Medical Center of PLA General Hospital of China, Beijing, P. R. China

The coronavirus disease 2019 (COVID-19) outbreak that was first reported in Wuhan, China has spread globally, causing unprecedented health and economic damage [1]. Although its clinical manifestations are mainly fever and respiratory symptoms, in some cases, gastrointestinal (GI) symptoms were the first manifestations [2]. Here we present a case of an elderly patient with COVID-19 who developed massive GI bleeding during treatment.

A 79-year-old woman was admitted in PLA General Hospital of China with a 4-day history of fever. She had a clear history of exposure to her daughter, a confirmed COVID-19 patient. Her serum and respiratory specimens tested positive for 2019 novel coronavirus (2019-nCoV) by reverse transcription–polymerase chain reaction. Physical examination revealed a body temperature of 38.5°C, blood pressure of 138/88 mmHg, pulse of 98 beats per minute, respiratory rate of 20 breaths per minute, and oxygen saturation of 96% while the patient was breathing room air. The platelet count was 91 × 10^9^/L and the prothrombin time was 16.1 seconds. Upon admission, she was alert and complained of fatigue, whole-body soreness, cough, expectoration, and occasional chest tightness. She was given recombinant human interferon α-2 b aerosol inhalation, oral lopinavir/ritonavir tablets, and moxifloxacin. Repeat chest X-ray examination showed that her pneumonia had worsened. Then the patient was transferred to the intensive care unit (ICU) with endotracheal intubation and mechanical ventilation, and was treated with methylprednisolone sodium succinate at the maximum dosage of 240 mg every 12 hours for type I respiratory failure. On the sixth day after admission, the dose of methylprednisolone was reduced and the patient was extubated on the eighth day. However, she developed several episodes of passing maroon-colored stools, with a volume of 350–500 mL each episode and blood pressure of 86/58 mmHg. Therefore, emergency bedside upper endoscopy and colonoscopy were performed. Because per-oral endoscopy can generate aerosol, all endoscopy staff were required to adopt level III protection according to the National Health Commission’s requirements on the prevention and control of COVID-19, which include disposable caps, medical protective masks (N95), positive-pressure headgear, disposable medical protective clothing, double-layer double-color latex gloves, disposable boot covers, and disposable shoe covers. The upper endoscopy was negative. During colonoscopy, fresh blood was noted throughout the colon and a few small diverticula (Figure 1). With a gradual improvement in the pneumonia, the dose of prednisolone was gradually decreased. A proton pump inhibitor was used to reduce a stress ulcer and oral mucosal protectant used to promote mucosal healing. With supportive care, the gross GI bleeding stopped within 3 days with a blood transfusion and the application of a carrol sodium sulfonate sodium chloride injection, human prothrombin complex, and recombinant human thrombopoietin.

**Figure 1.**
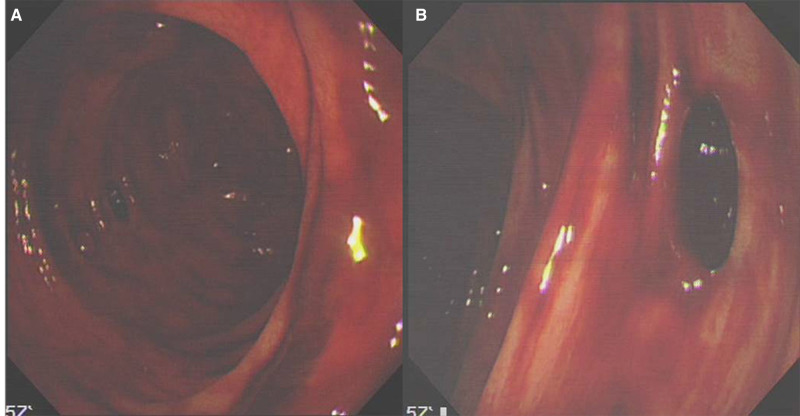
The colonoscopy presents fresh blood throughout the colon (A) and a few diverticula (B)

2019-nCoV virus exhibits high infectivity. In fact, many COVID-19 patients are asymptomatic. The main routes of virus transmission are through respiratory droplets and direct contact. However, it has been reported that viral nucleic acid testing of stool specimens and rectal swabs can be positive [1, 3]. Guan *et al.* [3] reported that some of the infected patients exhibited GI symptoms, including diarrhea (3.7%) and vomiting (5.0%), and that the incidence of increased levels of transaminases was 21%–22%. Angiotensin converting enzyme II (ACE2) is known to be a cell receptor for Covid-19 [4]. ACE2 protein is expressed not only on the lung, but also in the small intestine, colon, and liver [5, 6]. This suggests that the digestive system is also a target organ of COVID-19. Our patient with severe COVID-19 developed hematochezia and hemorrhagic shock due to bleeding. The authors suspect that the etiologies for her GI bleeding could be those as follows. (i) A viral direct cytotoxic effect in the gut. SARS-CoV-2 shares >85% homology with bat severe acute respiratory syndrome (SARS)-like coronavirus [1]. GI bleeding in patients with SARS is caused by the SARS virus itself, which leads to vasodilation and hyperemia of the GI mucosa and bleeding in specific GI segments [7, 8]. A previous study has reported that a patient severely COVID-19-infected with 2019-nCoV also exhibited coagulopathy [1]. It is possible that the severe condition of the patient was due to the direct action of 2019-nCoV on the target organ that led to coagulation abnormalities. (ii) ICU stay, steroid therapy, and mental stress can induce acute GI bleeding with excessive platelet consumption and the weakening of the self-protection mechanism [9]. (iii) Colonic diverticular bleeding might be induced by stress and viral infection. We hope that this case could add some additional information in the management of COVID-19 patients.

## Authors' contributions

Y.L.W. drafted the manuscript, and analysed and interpreted the data. W.H.Z. conceived and designed the project. J.S.M. and X.B.Q. collected the data. All authors read and approved the final manuscript.

## Funding

Not applicable.
